# Arsenic biotransformation potential of six marine diatom species: effect of temperature and salinity

**DOI:** 10.1038/s41598-019-46551-8

**Published:** 2019-07-15

**Authors:** Rimana Islam Papry, Kento Ishii, M. Abdullah Al Mamun, Sohag Miah, Kanako Naito, Asami S. Mashio, Teruya Maki, Hiroshi Hasegawa

**Affiliations:** 1 Graduate School of Natural Science and Technology, Kanazawa University, Kakuma, Kanazawa 920-1192 Japan; 2 Present Address: Department of Soil Science, Hajee Mohammad Danesh Science and Technology University, Dinajpur, Bangladesh; 3 Present Address: Institute of Forestry and Environmental Sciences, University of Chittagong, Chattogram, 4331 Bangladesh; 4 Department of Environmental Science, Faculty of Life and Environmental Sciences, Prefectural University of Hiroshima, Shibazuka-cho, Ebara-shi, Hiroshima, 727-0023 Japan; 5 Institute of Science and Engineering, Kanazawa University, Kakuma, Kanazawa 920-1192 Japan

**Keywords:** Biochemistry, Marine chemistry

## Abstract

Temperature and salinity effects on marine diatom species growth has been studied extensively; however, their effect on arsenic (As) biotransformation has been imprecise. This study reports the growth, and As biotransformation and speciation patterns at various temperatures and salinities of six marine diatom species: *Asteroplanus karianus*, *Thalassionema nitzschioides*, *Nitzschia longissima*, *Skeletonema* sp., *Ditylum brightwellii*, and *Chaetoceros didymus*. The growth rate and As biotransformation potentials of these species during three weeks of culture in f/2 based medium were significantly affected by wide temperature (0–35 °C) and salinity (0.3–50‰) ranges. Growth and As biotransformation were higher at optimum temperatures of 10–25 °C, and salinity of 10–35‰, whereas growth and arsenic biotransformation were lower at <5 °C and 5‰ and >25 °C and 35‰, respectively. The results showed that As(V) to As(III) biotransformation differed significantly (*p* < 0.05) between day 10 and 17. At optimum temperature and salinity levels, the cell size and As biotransformation were higher for all the species. A conceptual model on temperature and salinity effects on growth and As uptake and biotransformation mechanisms by these species has been proposed based on the findings of this study.

## Introduction

Arsenic (As), a toxic metalloid, is mostly found in soil, freshwater, and marine ecosystems. Anthropogenic activities, together with natural sources, contribute to increased contamination of surface and ground water. As has four oxidation states, such as arsenate (As(V)), arsenite (As(III)), arsenic (As°), and arsine (As(-III)), each of which comprises different physico-chemical characteristics^1^. Biotransformation of As species by aquatic organisms is a complex mechanism with different toxicity levels^2^. The toxicity of different As forms as determined by the 50% lethal dose (LD50) follow the order: As(III) (14) > As(V) (20) > monomethylarsonate (MMAA(V)) (700–1800) > dimethylarsinate (DMAA(V)) (700–2600) > arsenocholine (ArsC) (>6500) > arsenobetaine (ArsB) (>10,000)^3^. Inorganic arsenic (iAs) is more toxic than organoarsenic (orgAs); however, the toxicity on aquatic organisms depends on the As concentration and its speciation^4^. Microalgae reduces the toxic effect of iAs through several processes, including As(V) reduction, As(III) oxidation, methylation, conversion to arsenosugars/arsenolipids, complex formation of As(III) with glutathione and phytochelatins, cell surface binding, and excretion from the cell^5^. In marine environments, algal species uptake iAs (As(V) and As(III)) in the form of arsenic and biotransform it into methylated arsenic (methylAs) species and/or arsenosugars (AsS), such as orgAs species^6^. The concentration of iAs and methylAs species varies in marine microalgae cells, whereas As biomethylation produces methylAs species from iAs. This phenomenon is determined by the type of microalgae species and their characteristics because different species have various biotransformation abilities^7^. In the aquatic food chain, micro or macro algae, as members of lower trophic levels, take up As content more actively than higher trophic members^8^. According to Sanders *et al*.^9^, microalgae, as primary producer, accumulate As(V) from surrounding sea water and reduced it to As(III), and this biotransformation process elucidates the As(III) and As(V) ratio in marine aquatic environments. The chemical formation of As(III), MMAA) and dimethylarsinate (DMAA) are actively associated with primary productivity^10^ in marine water where microalgae play a pioneer role in the formation of such As species^11^.

However, growth of microalga, particularly diatom species, and their As biotransformation mechanisms are influenced by several factors, including constituency of nutrient medium^12^, concentration of As species^2,13^, pH^14–16^, light intensity^2,17^, temperature^18,19^, salinity^20,21^, and length of exposure period^22^. In this study, we focused on the two essential environmental factors, temperature and salinity, which regulate the growth and physicochemical metabolism in microalgae in marine ecosystems. The nutritional properties of microalgal species are stimulated by salinity and temperature variations in the environment^23^. Moreover, temperature plays a significant role on the growth rate^24^, chemical composition^25^, and metabolic processes of marine diatom species. Biochemical and physiological metabolism, such as growth, photosynthesis, and As accumulation and biotransformation are influenced by salinity in marine ecosystems. Although, adaptation and tolerance of microalgae varies between species, diatoms are directly or indirectly affected by salinity where the ion composition is one of the growth factors^26,27^. Many algal species can grow in a wide range of temperatures and salinities, exhibiting high tolerance to variation of such factors^28^.

The influence of temperature^25,29–32^ and salinity^33–35^ and their combined effect^36,37^ on the growth and development of marine diatoms have been studied in detail. However, limited information on the effect of temperature and salinity on As bioaccumulation, biotransformation, and speciation pattern, particularly by marine diatom species, is available. Moreover, the effects of salinity and temperature on cell sizes of marine diatom species and their interrelated influence on As biotransformation is also very limited in the literature. Therefore, this study aims to breech the research gap by investigating the influence of temperature and salinity on As biotransformation and speciation pattern by six marine diatom species. The interrelated influence of temperature, salinity, and cell size on As biotransformation was investigated. This study also highlighted the diatom species specifically associated with temperature- and salinity-dependent As biotransformation mechanisms, which may further provide important insight regarding As remediation processes.

## Results and Discussion

### Effects of temperature and salinity on the growth of marine diatom species

**Figure 1:**
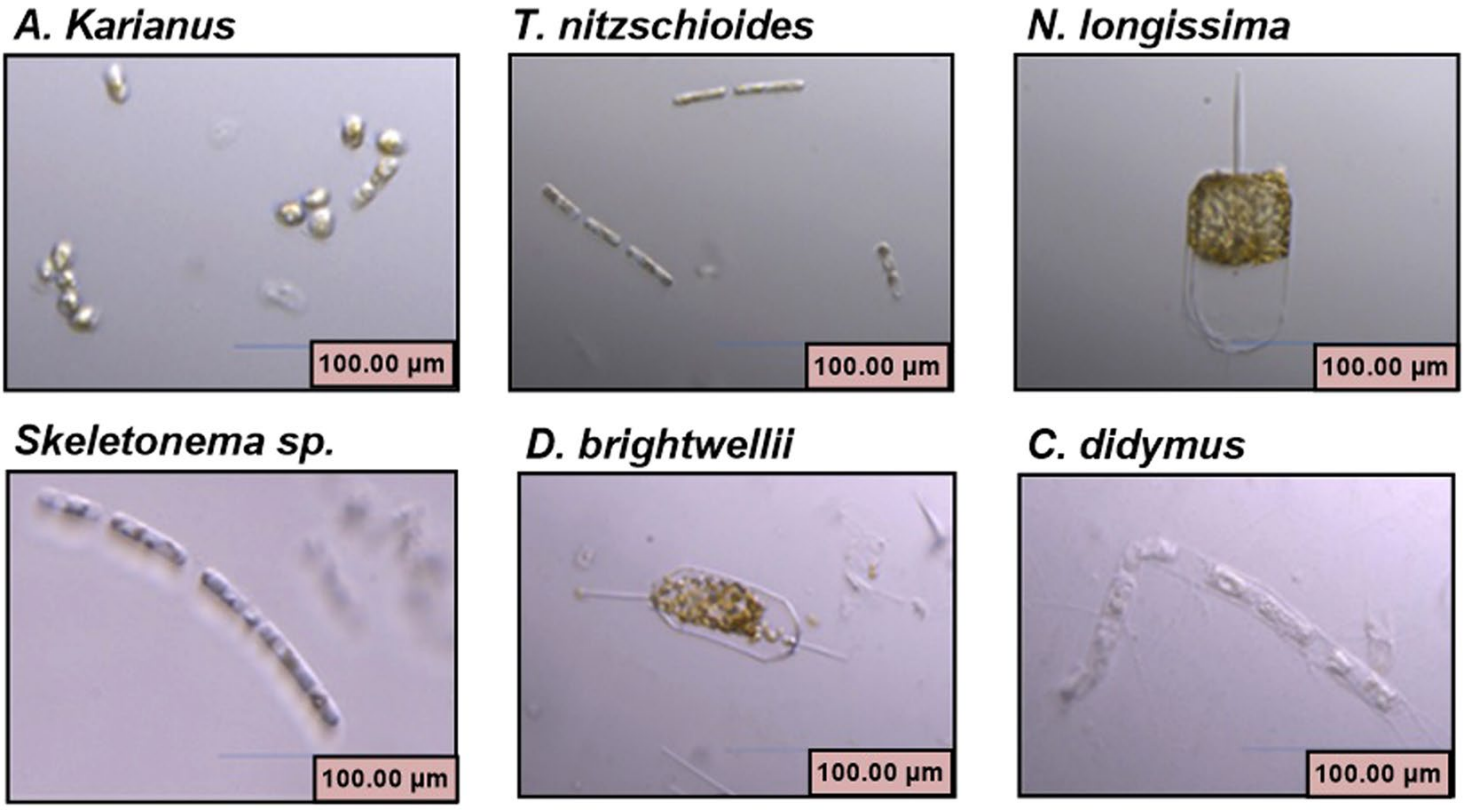
Images of six marine diatom species used in this study, captured using a digital microscope (KEYENCE, VHX-1000, Japan).

**Figure 2:**
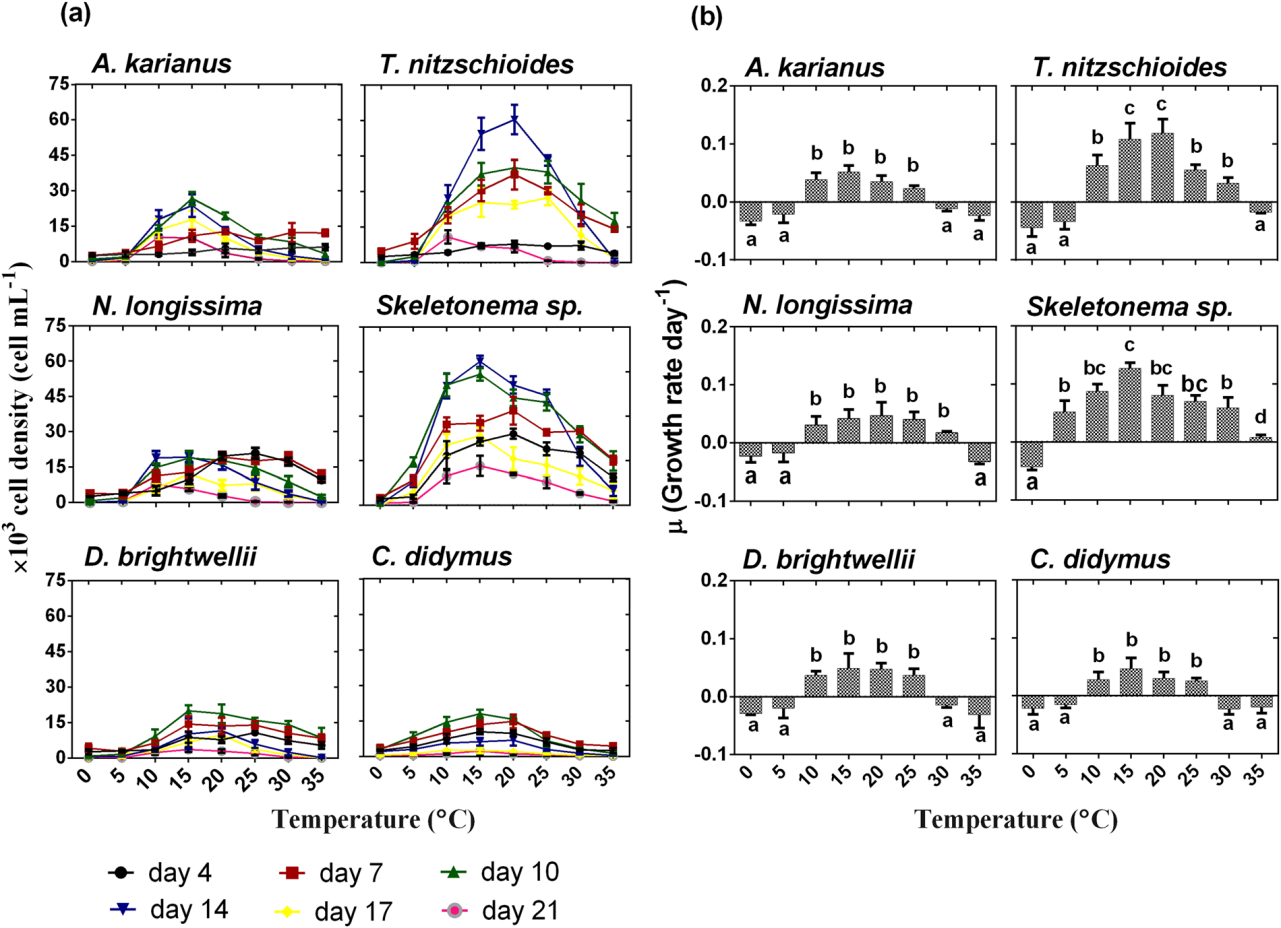
Effect of temperature (°C) on the growth of marine diatom species, (**a**) cell density (cell mL^−1^), (**b**) growth rate day^−1^. Different lowercase letter in Fig. 2(b) indicates significant differences between temperature levels. Data are means ± SD (*n* = 3).

Temperature is a factor that can be controlled in microalgal cultivation and is a sensitive factor for algal growth and metabolic processes. Digital microscope images of six marine diatom species used in this study are shown in Fig. 1. The maximum cell density (cells mL^−1^) was recorded at temperatures between 10 and 25 °C for all the species (Fig. 2a). Among the six species, *Thalassionema nitzschioides* and *Skeletonema* sp. showed exceptional cellular growth capacities with wide temperature tolerances. All the species exhibited higher cell densities on day 10 and 14 during the 3 weeks of culture. On day 14, the maximum cell concentration was recorded for *T. nitzschioides* (cell density 60.4 × 10^3^ cell mL^−1^/growth rate 0.11 ± 0.02 day^−1^ at 20 °C) and *Skeletonema* sp. (cell density 64.6 × 10^3^ cell mL^−1^/growth rate 0.12 ± 0.01 day^−1^ at 15 °C) (Fig. 2a,b). This result possibly suggests that these temperatures are optimum for the growth of these two species. Except for *T. nitzschioides* and *Skeletonema* sp., the remaining diatom species showed a similar growth pattern at various temperatures on different culture days. On day 10 at 15 °C, *Asteroplanus karianus*, *Nitzschia longissima*, *Ditylum brightwellii*, and *Chaetoceros didymus* displayed higher cell growth with cell densities of 26.8 × 10^3^ (growth rate 0.05 ± 0.01 day^−1^), 18.8 × 10^3^ (growth rate 0.04 ± 0.02 day^−1^), 20.1 × 10^3^ (growth rate 0.05 ± 0.03 day^−1^), and 18 × 10^3^ cell mL^−1^ (growth rate 0.05 ± 0.02 day^−1^), respectively (Fig. 2a,b). The effects of temperature on algal species may vary depending on the species type, characteristics, and surrounding environment. Fujimoto *et al*.^18^ reported that the growth rate of a microalga (*Selenastrum capricornutum*) was higher when temperature was adjusted to 22 °C, whereas that of *Microcystis viridis* was higher at 30 °C. A positive correlation was observed between temperature and *Nannochloris oculata* growth in a temperature range of 20 to 25 °C^38^, below this temperature the growth rate decreased from 0.13 to 0.06 day^−1^ ^39^. The optimum temperature for *Scenedesmus* sp. growth was found to be between 20 °C and 40 °C^40–42^. Algal species, such as *C. vulgaris* grow well at 30 °C; however, when the temperature was increased to 35 °C, the growth rate decreased to 17% and the species died at 38 °C^39^. Therefore, the growth of our experimental species was dependent on the capability to survive in certain temperature ranges, which is optimum for their growth.

**Figure 3:**
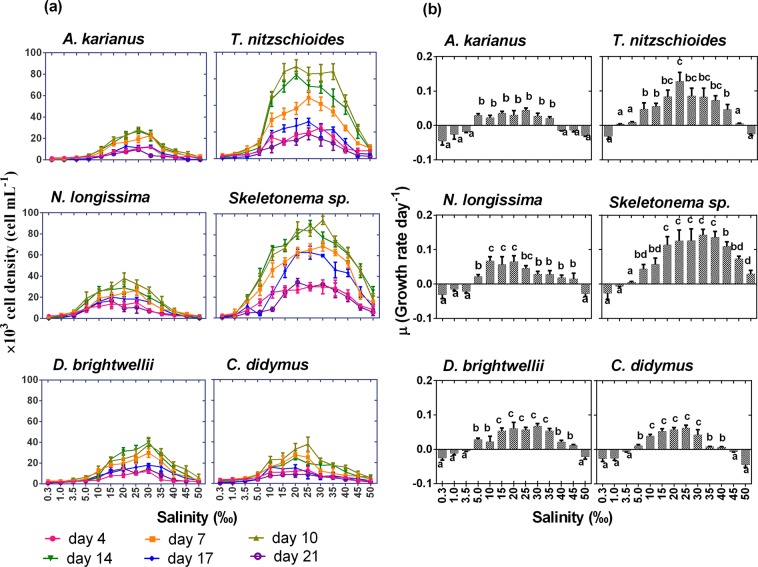
Effect of salinity (‰) on the growth of six marine diatom species, (**a**) cell density (cell mL^−1^), (**b**) growth rate day^−1^. Different lowercase letter in Fig. 3(b) indicates significant differences between salinity levels. Data are means ± SD (*n* = 3).

Salinity is an important factors that has significant effects on plant growth and their metabolic activities^43^. A wide range of salinity levels (0.3–50‰) was employed to observe the cell concentrations and growth rate per day of the marine diatoms in the culture vessels. All diatom species showed maximum cell concentrations on day 10 of incubation at salinities between 10 and 35‰ (Fig. 3a). Similarly, to the temperature treatment, *T. nitzschioides* and *Skeletonema* sp. showed higher salinity tolerances than the other experimental species. On day 10 of culture, the highest cell concentration of 69.2 × 10^3^ cell mL^−1^ (growth rate 0.13 ± 0.03 day^−1^) at 20‰ and 94 × 10^3^ cell mL^−1^ (growth rate 0.14 ± 0.12 day^−1^) at 30‰ salinity level was recorded for *T. nitzschioides* and *Skeletonema* sp., respectively (Fig. 3a,b). A similar maximum growth rate (0.06 ± 0.02 day^−1^) was observed for *N*. *longissima*, *D. brightwellii*, and *C. didymus*. A growth rate of 0.04 ± 0.01 day^−1^ with cell concentration of 28 × 10^3^ cell mL^−1^ was observed for *A. karianus* at 25‰ salinity level (Fig. 3a,b). Different salinity tolerance levels and adaptation strategies by microalgal species largely depend on the group and environmental characteristics^26^. In natural environments, multiplication of marine microalgae generally occurred between days 5 to 7 of their growth stage and they could survive for at least 21 days depending on the species. The growth and salinity tolerance (low growth expectancy between <5‰ and >35‰) of the marine diatom species of the present study were also supported by several previous studies^27,44,45^.

### Effects of temperature and salinity on As biotransformation

In this study, the cultures were exposed to 0.1 µmol L^−1^ Na_2_HAsO_4_ as As(V) and 1 µmol L^−1^ NaH_2_PO_4_ as PO_4_^3−^ to influence the uptake mechanisms of the diatom species. Microalgae take up arsenic species in the form of As(V) using the phosphate pathway in the cell membrane owing to the physiochemical similarities between As(V) and PO_4_ ^3–12,46^. Marine microalgae reduce the toxic effect of As(V) by transforming As(V) to As(III) inside the cell^47,48^. This phenomenon occurs by a reduction of two electrons of pentavalent arsenate to trivalent arsenite facilitated by thiol, such as glutathione^49^. The conversion of As(V) to As(III) and the following biomethylation to methylated arsenicals, e.g. DMAA and MMAA, by microalgae^50^ largely depends on the species growth capability and concentration of phosphate and arsenic in the culture medium.

**Figure 4:**
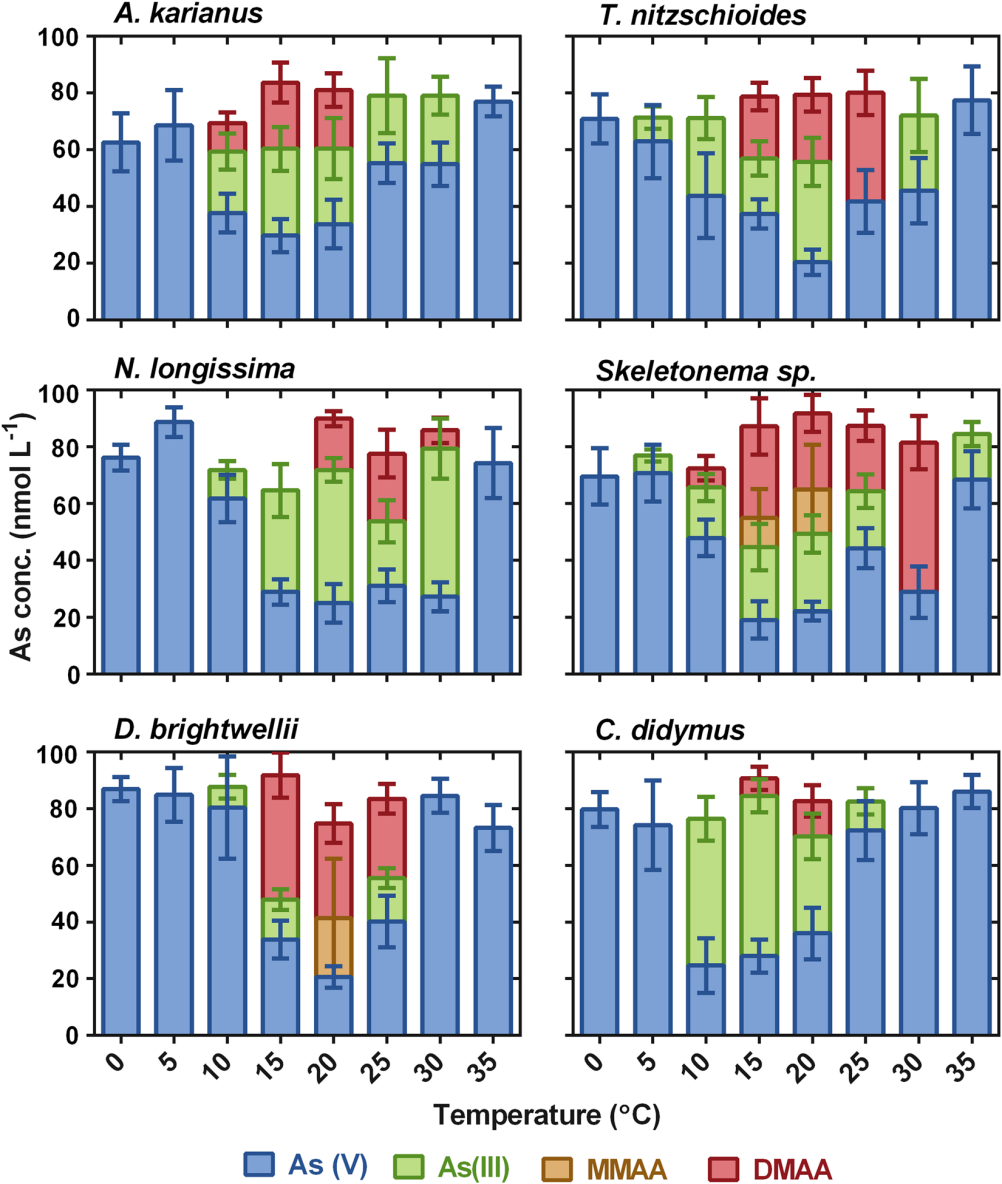
Effect of temperature (°C) on arsenic biotransformation processes (As(V) is reduced to As(III) and subsequent methylation to MMAA and DMAA) by six marine diatom species. Data are means ± SD (*n* = 3).

The potential of As biotransformation by six marine diatom species was investigated under various temperatures (0–35 °C) at day 10 of culture (Fig. 4). Except for *T. nitzschioides* and *Skeletonema* sp., none of the other microalgae showed biotransformation of As species at ≤5 °C and ≥35 °C. At 5 °C, As biotransformation was detected only for *T. nitzschioides* (62.9 ± 13 nmol L^−1^ of As(V) and 8.4 ± 4 nmol L^−1^ of As(III)) and *Skeletonema* sp. (As(V) = 73.7 ± 10 nmol L^−1^ and As(III) = 6.3 ± 2.1 nmol L^−1^), whereas *Skeletonema* sp. even biotransformed As at 35 °C (As(V) = 68.4 ± 10.1 nmol L^−1^ and As(III) = 16.1 ± 4.3 nmol L^−1^) (Fig. 4). All the species transformed As(V) to As(III) and the methylated forms of As species i.e. DMAA and/or MMAA at 15 and 20 °C. The reduction of As(V) to As(III) and/or DMAA was witnessed from 10–30 °C for *A. karianus* and *N. longissima*, 5–30 °C for *T. nitzschioides*, and 10–25 °C for *D. brightwellii* and *C. didymus* (Fig. 4). This phenomenon was observed for *Skeletonema* sp. even at a wide temperature range (5–35 °C). The above results indicated that all the diatom species of this study could grow and potentially biotransform toxic As(V) to As(III) with further methylation to form methylAs species at certain temperature ranges at their logarithmic growth phase.

The present study elucidated the relationship between temperature on the growth of diatom species and As biotransformation. None of the species showed biotransformation at 0 °C, suggesting that at such low temperatures these species are unable to reduce As(V) to As(III) and the subsequent methylation to MMAA and DMAA. However, the presence of small amounts of MMAA and MMAA not only assist in iAs methylation, but also help the fast release of MMAA and DMAA when exposed to As(V)^51^. Similarly, at 35 °C, no As biotransformation occurred, except for *Skeletonema* sp. for growth and As biotransformation processes. However, As(V) was reduced to As(III) either for further methylation or efflux from the cell^52^ by *D. brightwellii* and *C. didymus* at 10–25 °C. The influence of temperature on As uptake mechanisms and speciation were very imprecise. However, several researchers exposed cultures to temperatures of 20–35 °C to investigate As uptake and metabolism by phytoplankton^18,22,53^, suggesting that high temperatures are important to reduce arsenic uptake. Cho *et al*.^54^ observed that maximum cellular growth of *Chlorella ellipsoidea* and *Nannochloris oculata* at 20 °C and 30 °C, respectively, but the study did not investigate the As uptake and biotransformation processes. These findings illustrated that As biotransformation depends on the specific species and their metabolism, in addition to adaptability to different temperature conditions.

**Figure 5:**
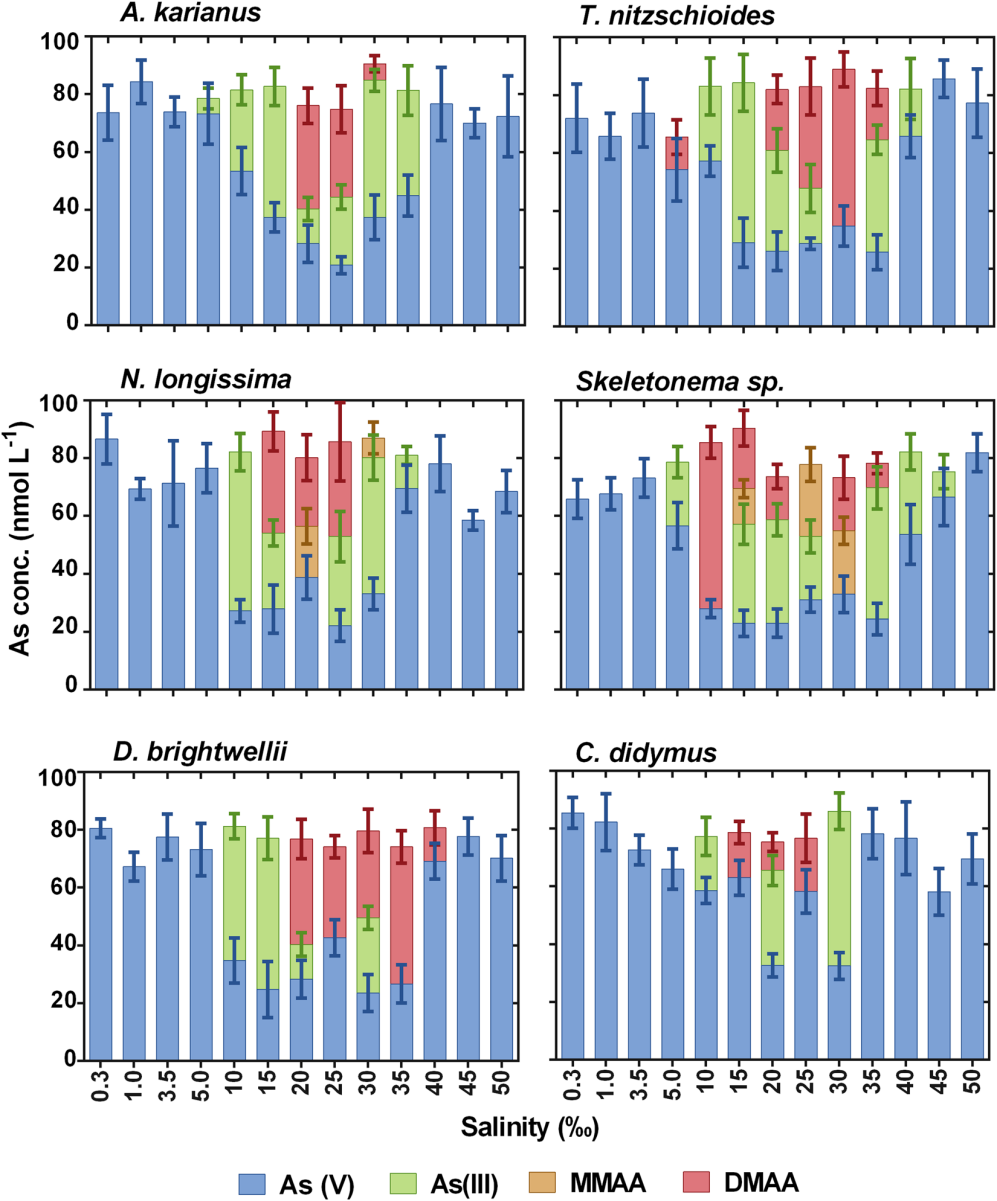
Effect of salinity (‰) on arsenic biotransformation processes (As(V) is reduced to As(III) and subsequent methylation to MMAA and DMAA) by six marine diatom species. Data are means ± SD (*n* = 3).

The biotransformation of As species and the subsequent methylation by six diatom species occurred well between salinities of 10 to 35‰ (Fig. 5). At low salinity levels (0.3–3.5‰), only As(V) was measured in the culture medium, indicating that at such low salinities all the species were unable to reduce As(V) to As(III) or methylated arsenicals. A 5‰ salinity was too low for cellular growth and As biotransformation of *N. longissima*, *D. brightwellii*, and *C. didymus*. At high salinity levels (45–50‰), no biotransformation was occurred, except for in *Skeletonema* sp. This species possesses a wide range of salinity tolerance (5–45‰) and could biotransform As(V) to As(III), even at a salinity of 45‰ (As(V) = 66.5 ± 9.8 nmol L^−1^ and As(III) = 8.8 ± 5.8 nmol L^−1^) (Fig. 5). This is might be because different species react differently in different salinities, which has been reported in several other studies^44,45,55^. This is an agreement a previous study indicating that *Skeletonema* sp. grow well under wide salinity conditions in the natural environment^34,56,57^. However, *C. didymus* showed As biotransformation under salinities between 10–30‰, whereas below 5‰ and above 35‰, the redox reaction, as well as methylation, was absent. This phenomenon may occur because (i) microalgal metabolism is affected by extreme high or low ion concentrations and (ii) certain metabolites required for cellular growth are depleted. In addition, salinity changes influenced changes in turgor pressure, which in turn modified the membrane thickness. The biochemical and biophysical processes of microalgal cells are possibly connected with the translocation rate of mobile charges located inside the membrane, which is regulated by the membrane thickness^58^. The redox reaction by microalgae generally occurs as a protection mechanism to avoid metalloid toxicity^14^, or as a supportive system for cellular growth in the mode of energy production^59^. Biotransformation activity by marine species may be influenced by different salinity conditions, as it has an obstructive effect on the central metabolic activity^43^.

### Effects of temperature and salinity on As speciation pattern: time dependency study

**Figure 6:**
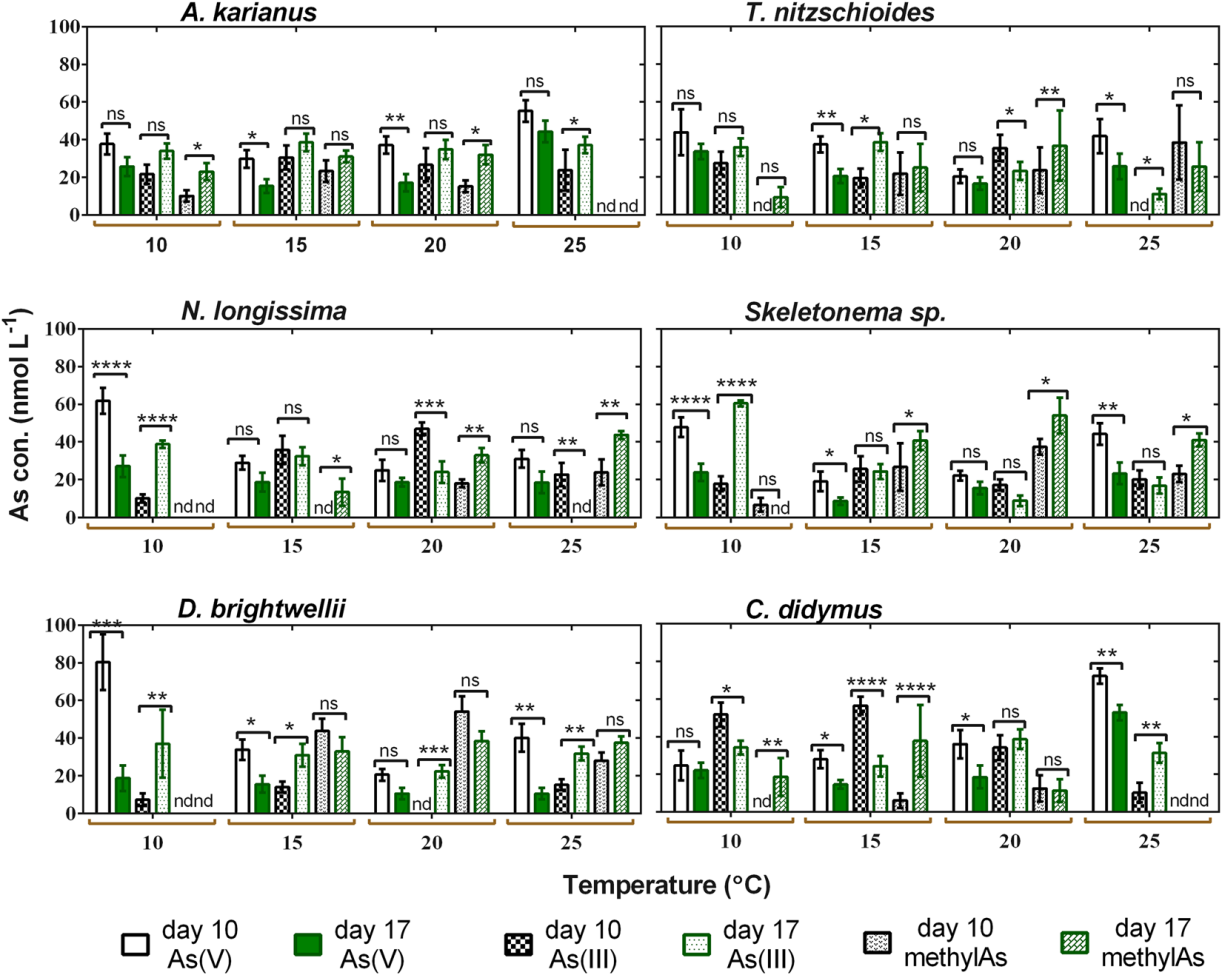
Time-dependent As speciation patterns by six marine diatom species. As speciation was observed on day 10 and 17 of culture at various temperatures (°C). The star marks above the bars showed significant differences at *p ≤ 0.01, **p ≤ 0.001, and ***p ≤ 0.0001 levels between day 10 and 17 within the same As species. ‘ns’ indicates non-significant, ‘nd’ indicates not detected. Data are means ± SD (*n* = 3).

Arsenic speciation pattern by marine diatoms was observed at four different temperatures (10, 15, 20, and 25 °C) in a week i.e. on day 10 and 17 of culture (Fig. 6). These temperatures seemed optimum to all the species, as growth and As biotransformation were not properly observed for all the species below 10 °C and above 25 °C. The result indicated that in some cases, the biotransformation of As(V) to As(III) and subsequent methylated arsenicals was significantly different (*p* < 0.05) between day 10 and 17 of speciation. For example, on day 10 at 10 °C, *A. karianus* contained 37.7 ± 5.6, 21.7 ± 5.2, and 10.1 ± 3.1 nmol L^−1^ of As (V), As(III), and methylated As component (MMAA + DMAA), respectively, whereas on day 17 at the same temperature, these amounts were 25.7 ± 4.9, 33.9 ± 4.0, and 23 ± 4.4 nmol L^−1^ (Fig. 6). Regarding *T. nitzschioides*, the concentrations of As(V), As(III), and methylAs component were 43.8 ± 12.1, 27.4 ± 6.0, and 0 nmol L^−1^ on day 10 at 10 °C, whereas these amounts were 33.6 ± 4.0, 35.9 ± 4.8, and 9.5 ± 5.2 nmol L^−1^ on day 17 at the same temperature, respectively (Fig. 6). Other species, such as *N. longissima*, *Skeletonema* sp., *D. brightwellii*, and *C. didymus* showed significant As biotransformation from days 10 to 17. Lower As(V) concentrations on day 17 than on day 10 at each temperature indicated that As(V) decreased with time in the culture medium. The occurrence of As(III) and methylAs in the medium was mainly a biological reduction of As(V) to As(III). In addition, methylated arsenicals (MMAA and DMAA) were also found at higher concentrations on day 17 than that on day 10. The conversion efficiency of pentavalent arsenate (As(V)) to the reduced form of trivalent arsenite (As(III)) was considered to be the pioneer step for the methylation process^60^. As biotransformation by marine diatom species in this study revealed that they are capable of redox reactions, methylation, and excretion of As in the medium.

**Figure 7:**
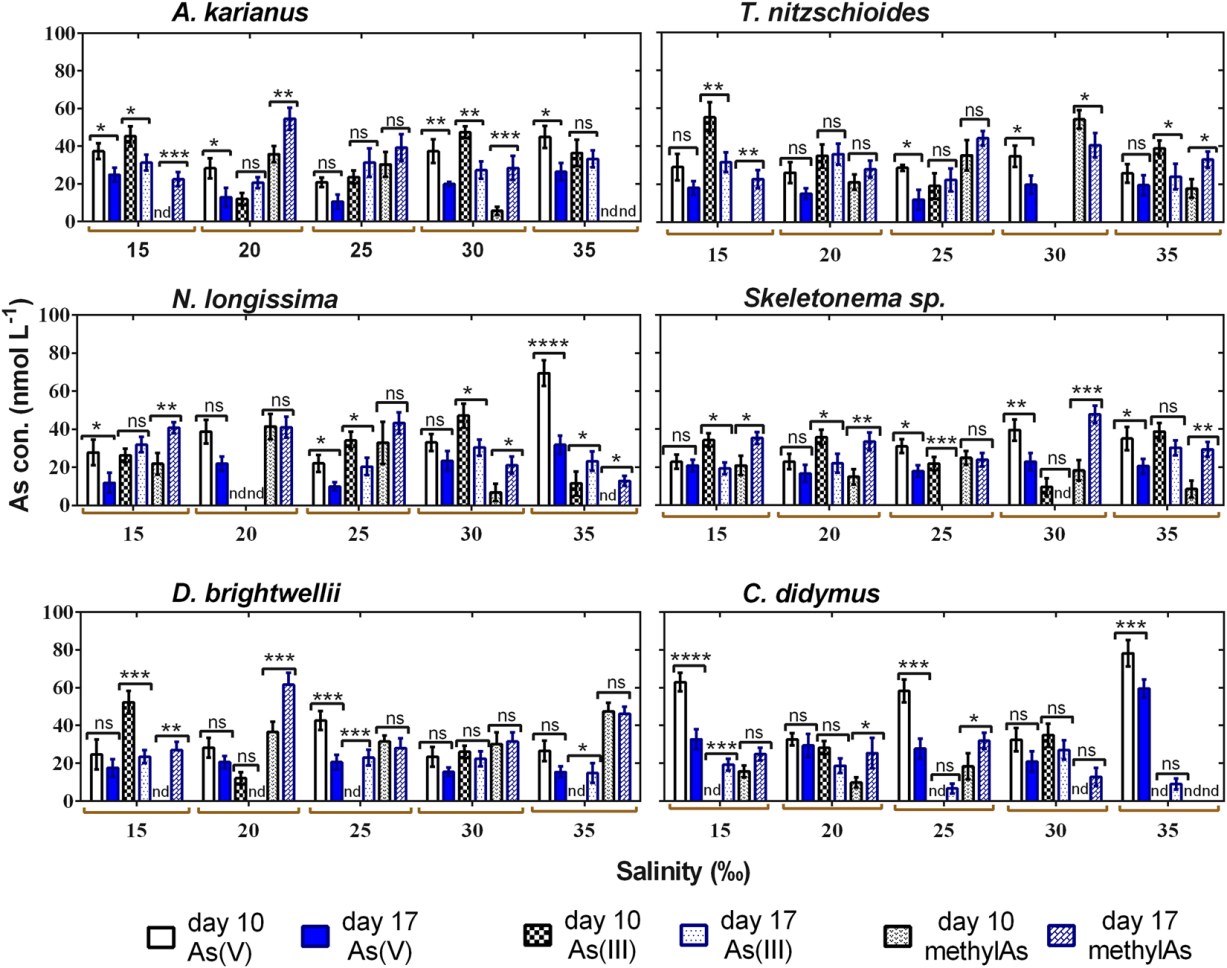
Time-dependent As speciation pattern by six marine diatom species. As speciation was observed on day 10 and 17 of culture at various salinity (‰) levels. The star marks above the bars showed significant differences at *p ≤ 0.01, **p ≤ 0.001, and ***p ≤ 0.0001 levels between day 10 and 17 samples within the same As species. ns indicates non-significant, nd indicates not detected. Data are means ± SD (*n* = 3).

Similarly, to temperature, a time dependency study was conducted at five salinity levels (15_,_ 20_,_ 25, 30, and 35‰), and As speciation was recorded on day 10 and 17. At all salinities, the biological reduction of As(V) by marine diatom species was recorded between day 10 and 17 (Fig. 7). For example, at day 10 and 25‰ salinity condition in *T. nitzschioides*, the concentrations of As(V), As(III), and methylAs were 28.6 ± 1.6, 19.1 ± 6.8, and 35.2 ± 8.0 nmol L^−1^, whereas on day 17 at the same temperature these concentrations were 11.9 ± 5.2, 22.2 ± 6.1, and 44.2 ± 3.9 nmol L^−1^, respectively (Fig. 7). In some cases, at certain salinity conditions on day 10, more than 60% As(V) remained in the medium. For example, on day 10 at 35‰ salinity, *N. longissima* and *C. didymus* species contained 69.4 ± 6.7 and 76.6 ± 7.0 nmol L^−1^ of As(V), respectively (Fig. 7). However, *C. didymus* contained an As(V) concentration of 59.5 ± 4.8 nmol L^−1^ at day 17 at 35‰, which was the only exception found during the analysis of the data related to salinity-based time dependency, but is difficult to explain. As the regulation of As uptake is different for different species, it may be controlled by the intracellular As(V) concentration, and then its accumulation could be activated by its release from the cell^5,61^. As(V) uptake or sequestration is enhanced by the physio-biochemical structure in association with individual species type and characteristics^62^.

### Interrelated influence of temperature, salinity, and cell size on As biotransformation

**Figure 8:**
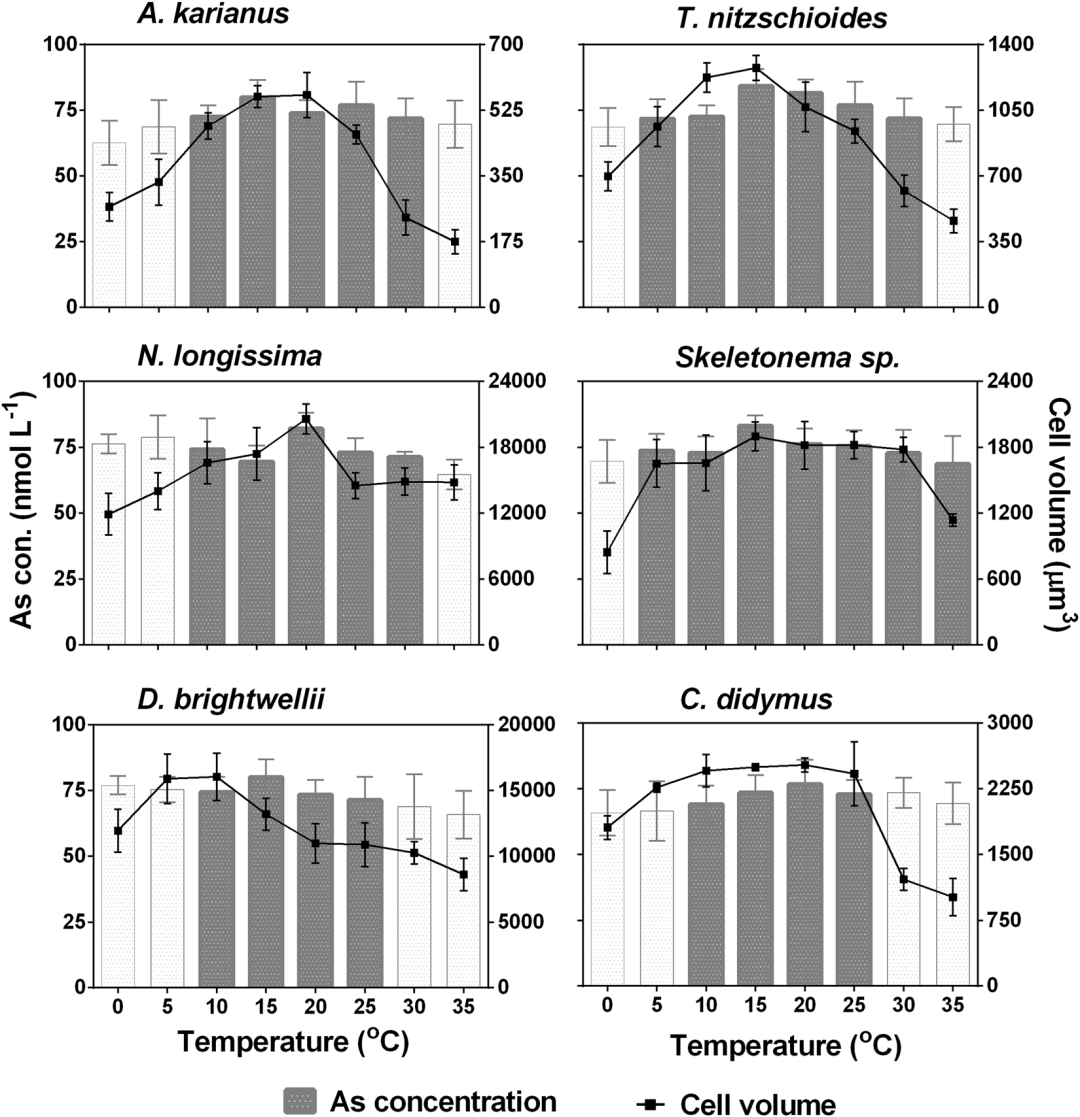
Relationship between cell size and As biotransformation of six marine diatom species at various temperature levels. Dark bars in the graph indicate As species biotransformation occurred, i.e. As(V) was reduced to As(III) and the subsequent methylation to MMAA and DMAA. White bars indicate no biotransformation occurred, i.e. only As(V) was detected in the culture medium. Data are means ± SD (*n* = 3).

**Figure 9:**
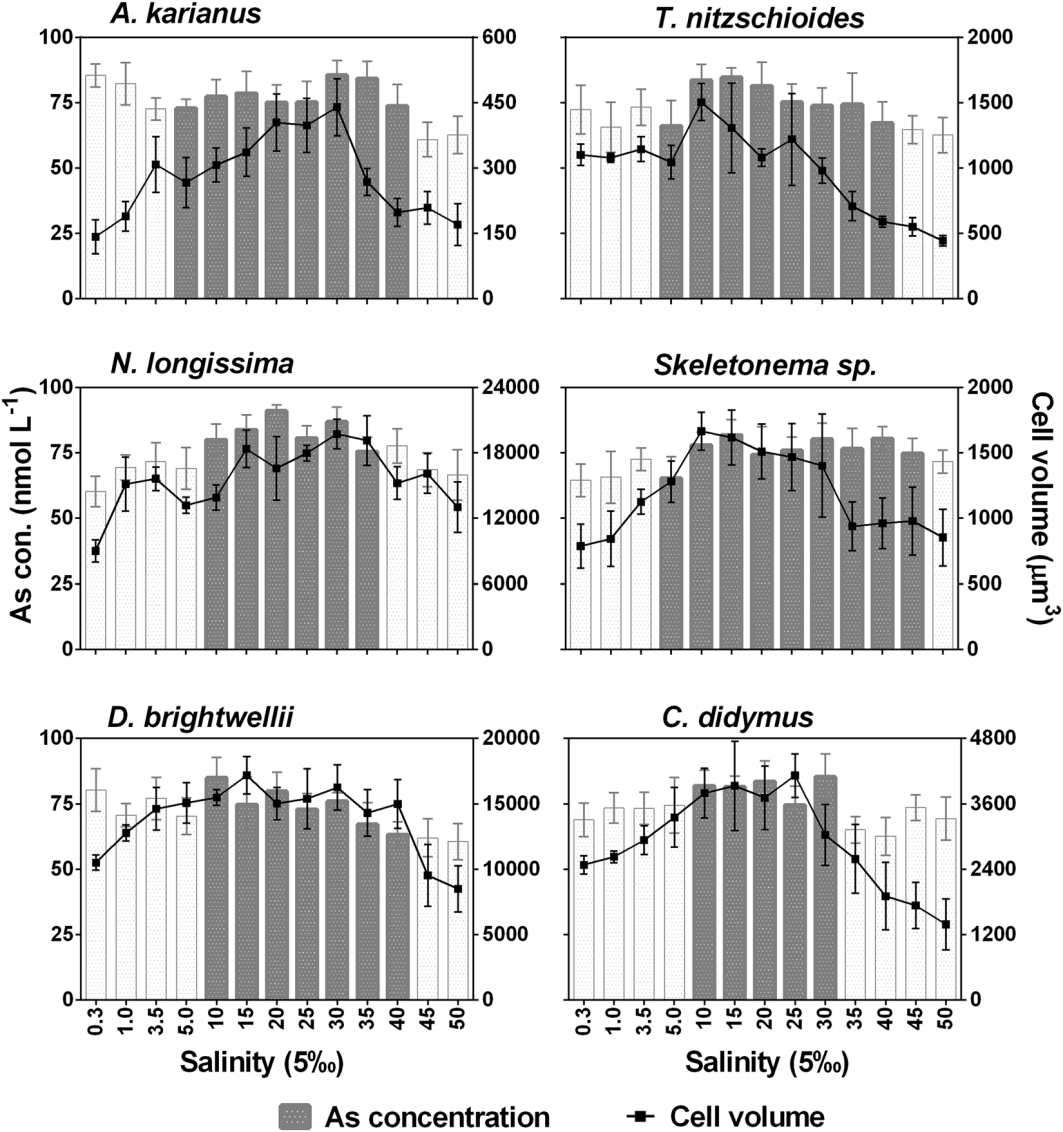
Relationship between cell size and As biotransformation of six marine diatom species at various salinity levels. Dark bars in the graph indicate As species biotransformation occurred, i.e. As(V) was reduced to As(III) and the subsequent methylation to MMAA and DMAA. White bars indicate no biotransformation occurred, i.e. only As(V) was detected in the culture medium. Data are means ± SD (*n* = 3).

Cell surface area (µm^2^) of six diatom species was calculated at different temperatures (0–35 °C) and salinities (0.3–50%_o_) (Table S1, Appendix A: Supporting information). The maximum surface area of the cell was in the ranges of optimum temperature (10–25 °C) and salinity (10–35‰) for all the species. Below and above these temperature and salinity levels, individual diatom cells had a reduced surface area for adaptation to the adverse environment. The interrelation between cell volume (µm^3^) of each diatom species and As biotransformation potentials were evaluated (Figs 8, 9). The cell volume and As biotransformation were positively correlated in almost all of the cases. Cell size of the six diatom species in the descending order: *N. longissima* > *D. brightwellii* > *C. didymus* > *Skeletonema* sp. > *T. nitzschioides* > *A. karianus*. At the optimum temperature and salinity levels, all the species could biotransform As(V) to As(III) or MMAA or DMAA at a maximum cell volume. The diatom species were unable to biotransform As species at temperatures <5 °C and >30 °C, and salinity <5‰ and >35‰ with some variations (Figs 8, 9). The As biotransformation potentials of the six diatom species during temperature treatment was: *Skeletonema* sp. > *T. nitzschioides* > *N. longissima* > *A. karianus* > *D. brightwellii* > *C. didymus*, whereas during the salinity treatment the order was: *Skeletonema* sp. > *T. nitzschioides* > *A. karianus* > *D. brightwellii* > *N. longissima* > *C. didymus*.

### Conceptual model of growth and arsenic uptake mechanisms

**Figure 10:**
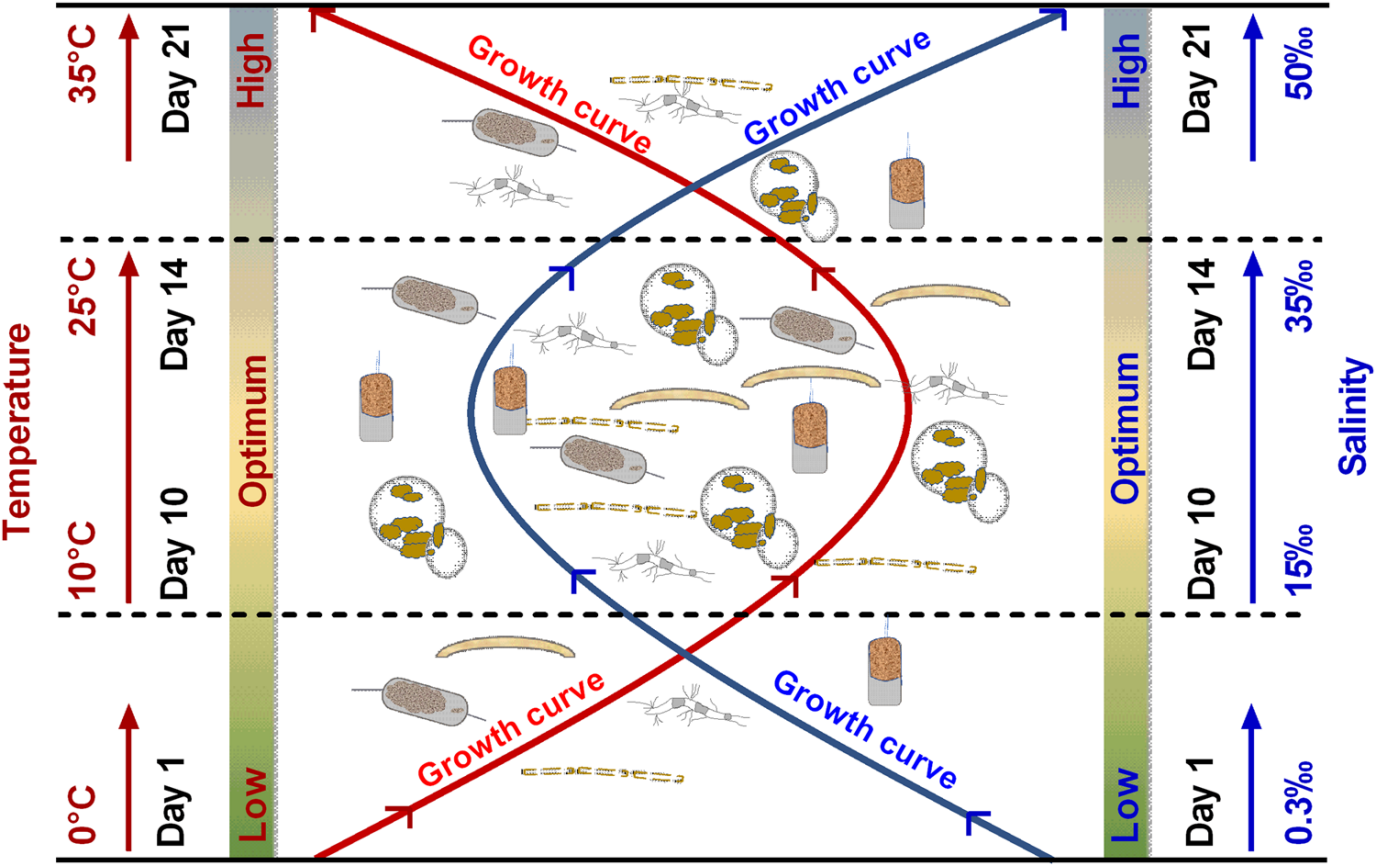
Conceptual model on effect of temperature and salinity on the growth of six marine diatom species.

**Figure 11:**
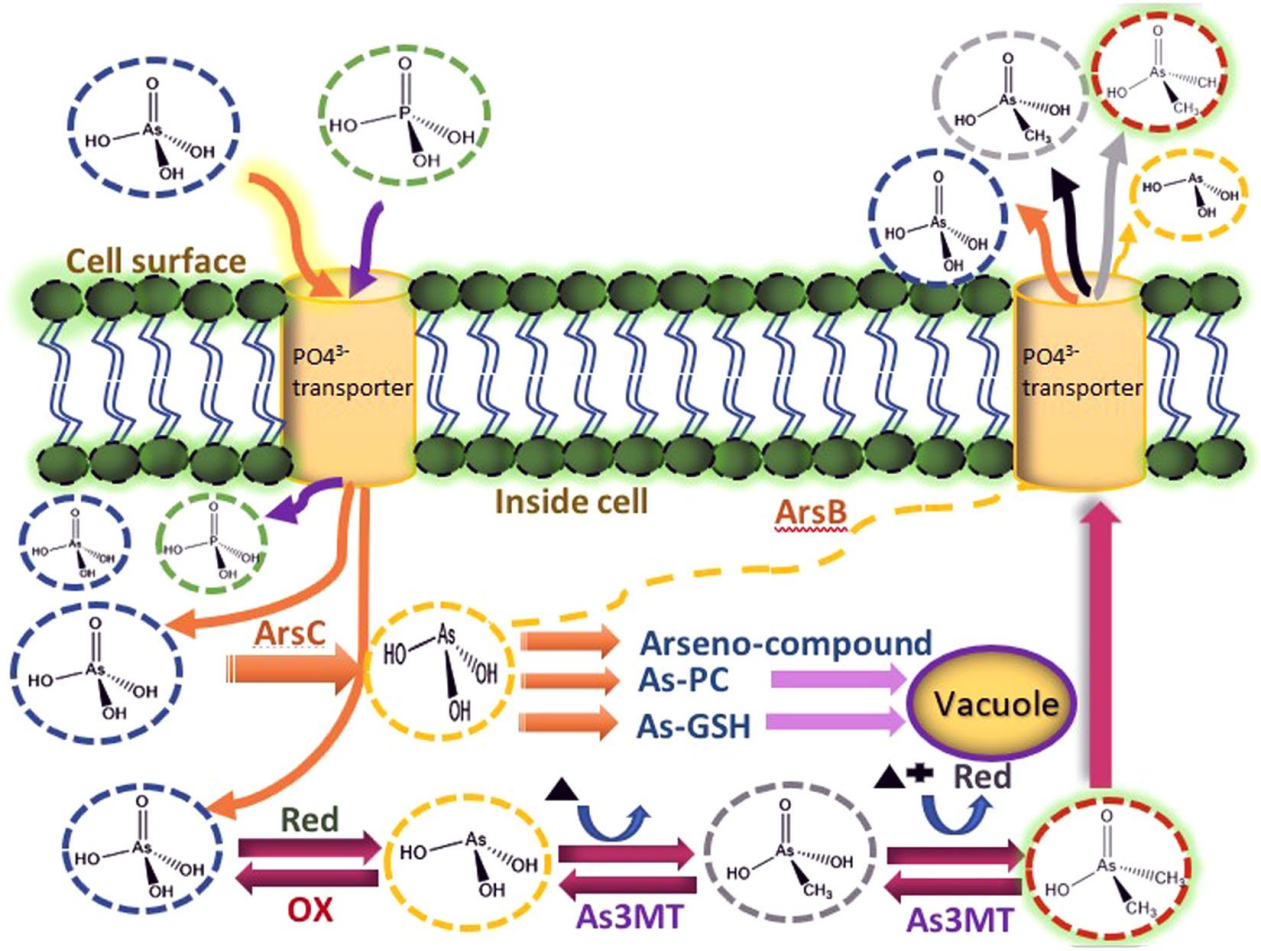
Conceptual model on effect of temperature and salinity on arsenic uptake and biotransformation mechanisms by six marine diatom species based on this study.

A conceptual model on the effect of temperature and salinity on the growth and As uptake and biotransformation mechanisms by six marine diatom species was developed based on the findings of this study (Figs 10, 11). Microalgal growth is influenced by environmental conditions, such as temperature and salinity^28,63,64^, which are considered as crucial environmental factors during their culture. The effect of both temperature and salinity on the growth of six marine diatom species showed similar trends based on the culture day. The maximum cell density (cell m L^−1^) was recorded for all the diatom species on day 10 and 14 of culture for both temperature and salinity treatments. All the species showed optimum growth as cell density at temperatures between 10 and 25 °C and salinity levels between 10 and 35‰ (Figs 2a, 3a, 10). The temperature tolerance of algal species depends on rapid changes of temperature and the physicochemical state of algae exposed to extreme temperature before, during, and after a period of time^65^. Moreover, salinity influences the growth of the species regulated by osmoregulatory mechanisms, which further affects the physiological and biochemical process of algal species in marine environments.

In aquatic environments, As(V) accumulation and biotransformation to As(III) and subsequent methylation to MMAA and DMAA by photosynthetic microbial species e.g. algae and cyanobacteria^50^, is affected by environmental factors, such as temperature, salinity, pH, and nutrients. These factors control the algal biochemical composition and behaviour in natural aquatic ecosystems. These microbial species play a significant role in regarding biogeochemical cycling of As content by biotransformation in natural ecosystems^48^. As(V) and phosphate have similar physicochemical characteristics, and therefore algal species take up As(V) present in media via a phosphate transporter system^66^ and As(V) biotransformation occurs inside the cell^67^. The competitive uptake between As(V) and PO_4_^3−^ suggested a possible active toxic mode of As. Inside the cellular portion of ATP, As(V) replaced the phosphate groups and established an unstable ADP-As complex that in turn interfered with several physiological process, e.g. energy flow^68^. Furthermore, after As(V) uptake, it is either transformed to trivalent As and excreted into the medium or subsequently methylated to methyl arsenicals. The reduced metabolites As(III) readily released from the cell leading to reduced the toxicity of As(III) inside of the cell^69,70^. This process of biomethylation from iAs is regarded as the detoxification mechanism of aquatic algal species^71^. In addition, excretion of As(V), As(III), or the organic component could be regarded as a significant arsenic detoxification mechanism^72^.

As(V) toxicity is based on the competitive inhibition of protein, along with other enzymes that use phosphate, as well as oxidative phosphorylation. The reduction reaction of As(V) to As(III) occurs in the presence of thiols and/or dithiols because of its tendency to combine with biochemical components, such as protein and non-protein thiols^73^. However, As(III) is more toxic than As(V) because of its actions, similarly to soft metal with thiols. In addition, the As(III) bond with monothiol is comparatively weak and its high concentration depletes glutathione within the cell. It develops strong bonds with dithiols, which inactivates different important enzymes and receptors^74^. After As(V) uptake from surrounding marine water, As(V) is organized to form carbohydrate groups, which are further biosynthesized to orgAs species^6^. However, orgAs contained by marine microalgae are mostly AsS that are regarded as pioneers in the metabolic channel to ArsB and ArsC^75^. ArsB acts as an antiporter that excludes As(III) from cells during the exchange by H^+^/As(OH)_3_ attached to the electrochemically mediated proton gradient^76^. ArsC is the substrate of ArsB that acts as an As(V) reductase. ArsC transforms As(V) to As(III) and increases the array of resistance to include As(V)^77^.

The biotransformation process of As(V) to As(III) takes place via aquaporin nodulin 26-like intrinsic protein (NIPs)^72^, which is a water channel that transports water molecules from extracellular to intracellular portions. This reduction reaction occurs in the presence of several reductases that act as electron donors, such as glutaredoxin, glutathione, or thioredoxin^78^. As an electron donor, glutathione reduces As(V) to As(III) in aqueous solution and forms a complex of arsenotriglutathione, As(III) (GS)_3_ that immediately gives As(III) to target species that contain dithiol groups^79^. Methylation mechanisms by the As(III) (GS)_3_ complex has been discussed in the study of Hayakawa *et al*.^80^.

Phytochelatins (PCs), with metal binding capacities, are intracellular thiol-based polypeptides of microalgae. These metalloproteins play a significant role in microalgal detoxification mechanisms. PCs encourage As to bind with thiol groups, i.e. glutathione has a significant function in As complexation and detoxification^16^. It has been suggested that even at lower As concentrations, the rate of PC synthesis is sufficient to bond with As and cells of marine microalgae species^81^. The reduction mechanism of As(V) to As(III) is a rapid and important process for further sequestration into the vacuoles of algal cells^82^ where the majority of As(V) content is reduced within 1–2 days prior to sequestration into the vacuole^71,73^.

In the present study, the As(III) concentration gradually decreased owing to continuous abiotic oxidation indicating that As(V) constrained the intracellular As(III) accumulation in algal cells^69^ (Fig. 11). Further, methylation of As(III) to methylated species (MMAA and DMAA) occurred within the cells and their overall concentration gradually increased in the growth medium by their excretion from the cell. However, there are several steps associated with the biochemical metabolism of inorganic As to methylated arsenicals, i.e. mono-di and tri-methyl arsenic species. Some steps are activated by chemical reactions, whereas others are incorporated into enzymatic catalysis. Previously it was found that one catalyst, arsenic-methyltransferase (AS3MT), actively promoted the biotransformation of iAs to methylated arsenic species^82^. However, the methylation mechanism is an oxidative process where a methyl group (CH_3_^+^) is available for the reaction to progress. But, oxidative stress occurred whenever active oxygen come in contact during the catalytic reaction with methyltransferase^83^. During deviations in the redox state of arsenic, S-adenosylmethionine (SAM) donated methyl groups to As using AS3MT. For methylation mechanisms, SAM is required because the extent and pattern of methylation mechanisms largely depends on its availability^84^.

## Conclusion

The potential growth and As biotransformation by six marine diatom species was investigated under various temperature (0–35 °C) and salinity (0.3–50‰) conditions during three weeks of culture. Except for *T. nitzschioides* and *Skeletonema* sp., none of the species biotransformed As species at ≤5 °C and ≥35 °C. However, growth and As biotransformation and subsequent methylation were optimum between temperatures of 10 to 25 °C and salinities of 10 to 35‰. At low salinity levels (0.3–3.5‰), only As(V) was measured in the culture medium, indicating that at such low salinity conditions all the species were unable to reduce As(V) to As(III) or methylated arsenicals. The biological reduction, i.e. biotransformation of As(V) to As(III) and subsequent methylated arsenicals, was significantly different between day 10 and 17 speciation at different temperature and salinity conditions. The interrelated influence of temperature, salinity, and cell size on As biotransformation was also reported for the first time. These results suggest that each species has an optimum temperature and salinity tolerance range suitable for their adaptation metabolism, such as growth and the biotransformation of toxic As(V) to As(III) and further methylation to form methylated As species.

## Materials and Methods

### Marine diatom species

Six strains of marine diatom species, *A. karianus*, *T. nitzschioides*, *N. longissima*, *Skeletonema* sp., *D. brightwellii*, and *C. didymus*, were used in this study (Fig. 1). Diatom species were supplied by Dr. Kanako Naito, Associate Professor of Hiroshima Prefecture University, Japan. *Asteroplanus karianus* is a pennate diatom distributed in coastal waters globally, but there is limited information available on its growth, physiology, and life cycle. This strain was isolated in June 2014 from Tsugaru Strait, Hokkaido, Japan. *Thalassionema nitzschioides* is a yellow-brown pennate diatom with a wide range of salinity tolerance (12–38‰). *Nitzschia longissima* is a free-living single-celled organism, which is motile and attached to the soft substratum of marine macrophytes, especially on seagrass leaves. *Skeletonema* sp. is a centric, cylindrical diatom that can survive in water temperatures up to 30 °C and causes water discolouration. *Thalassionema nitzschioides*, *N. longissimi* and *Skeletonema* sp. strains were isolated in June 2015 from Nanaehama, Hokkaido, Japan. *Ditylum brightwellii* is a marine centric unicellular photosynthetic autotroph. This strain was isolated in March 1989 from Hiroshima Bay, Japan^85^. *Chaetoceros didymus* is a photosynthetic centric diatom that is connected in straight chains. This strain was isolated in June 2015 from Nanaehama, Hokkaido, Japan. The cell size and its effect on the biotransformation potentials were taken into consideration during the selection of these six species.

### Reagents

Deionized water (arium pro UV, Sartorius Stedim Biotech, Goettingen, Germany) with a resistivity of 18.2 MΩ was used for all experiments. Reagents were commercially available and used without further purification. Sodium hydroxide (NaOH; special grade, Nacalai Tesque, Kyoto, Japan) and hydrochloric acid (HCl; Kanto Chemical, Tokyo, Japan) were used for the pH adjustment of reagents and medium. 4-(2-hydroxyethyl)-1-piperazinyl ethane sulphonate (HEPES; Nacalai Tesque) was used as a buffer reagent in culture medium. Special grade sodium dihydrogen phosphate (NaH_2_PO_4_) and disodium hydrogen arsenate heptahydrate (As(V)), both from Wako Pure Chemical (Osaka, Japan), were used as the phosphate and arsenic sources, respectively, in the culture medium.

### Preculture and maintenance of marine microalgae

Marine diatom species were maintained in f/2 based nutrient medium (Table 1) in natural sea water. Culture medium and the apparatus (tips, bottles, vessels, micropipettes) were sterilized separately at 121 °C for 30 min in an autoclave (MLS 3780, Sanyo Electric, Japan), followed by UV irradiation for 20 min on a clean bench (NK Clean Bench, VSF-1300A, Nippon, Japan). Before using the diatom species in experiments, cultures were maintained in the same medium for 1–2 weeks in polycarbonate bottles (Nalgene, Nunc; Rochester, NY) until they reached an exponential growth phase in a temperature- and light-controlled incubator (Koitotron3HN-35MLA, Koito Industries, Japan).

**Table 1:** Chemical composition of modified f/2 culture medium in artificial seawater.

Chemicals	Concentration (M)
NaNO_3_	8.82 × 10^−4^
NaH_2_PO_4_·2H_2_O	3.84 × 10^−5^
Na_2_SiO_3_·9H_2_O	3.46 × 10^−5^
Na_2_SeO_3_	1.00 × 10^−7^
CoSO_4_·7H_2_O	4.27 × 10^−8^
ZnSO_4_·7H_2_O	7.31 × 10^−8^
MnCl_2_·4H_2_O	9.10 × 10^−7^
CuSO_4_·5H_2_O	2.80 × 10^−8^
Na_2_MoO_4_·2H_2_O	2.89 × 10^−8^
Vitamin B_12_	3.69 × 10^−10^
Thiamine HCl	2.96 × 10^−7^
Biotin	2.05 × 10^−9^

### Growth and As speciation under various temperature and salinity conditions

The growth and As speciation of diatom species were observed at different temperatures and salinities. For the temperature treatment, diatom cultures were placed into polycarbonate vessels with 30 mL of sterilized culture medium containing natural sea water. The vessels for each batch of culture were kept in an incubator and set at a certain temperature (0, 5, 10, 15, 20, 25, 30, and 35 °C). Artificial sea water (Table S2, Appendix A: Supporting information) at various salinities (0.3, 1.0, 3.5, 5.0, 10, 15, 20, 25, 30, 35, 40, 45, and 50‰) was used to culture the diatom species for the salinity treatment. After incubating the diatom species, As(V) (0.1 µmol L^−1^) and PO_4_^3−^ (1 µmol L^−1^) were added to the culture medium. The cultures were grown for three weeks. The liquid samples were collected at day 4, 7, 10, 14, 17, and 21, and filtered using 0.45 µm cellulose membrane filters (Toyo Roshi Kaisha, Tokyo, Japan). Growth of each species was measured spectrophotometrically using a UV-VIS spectrophotometer at 540 nm and was calculated with an established cell density-to-absorbance ratio to estimate cell number. Cell numbers were counted using a digital microscope (Keyence, VHX-1000, Japan). The initial cell concentration of the diatom species was measured as 2.4 × 10^3^ cell mL^−1^. Growth rate per day was calculated using Eq. (1) ^64^:

1 $$\mu ({{\rm{day}}}^{-1})=\frac{(\mathrm{ln}\,{N}_{1}-\,\mathrm{ln}\,{N}_{0})}{t}$$

where, N_1_ = final cell density, N_2_ = initial cell density, and t = time (day).

### Arsenic speciation analysis

Arsenic species in culture media samples were determined using a hydride generation technique according to Hasegawa *et al*.^86^. A flame atomic absorption spectrophotometer (AAS) combined with hydride generation device followed by cold trapping (AAS, 170-50A, Hitachi, Japan) was used. Inorganic As (As(V) + As(III)), MMAA(V), DMAA(V) were analysed by adding 5.0 mL 0.20 mol L^−1^ EDTA·4Na (ethylenediaminetetraacetic acid; Kanto Chemicals) and 5.0 mol L^−1^ HCl to 40 mL of the sample solution. For As(III), 5.0 mL 0.20 mol L^−1^ EDTA·4Na and 0.5 mol L^−1^ potassium hydrogen phthalate (Kanto Chemicals) were added to 40 mL of sample solution. Arsenic species were recorded as a chromatogram on a data processing device (Chromato-PRO, Runtime Instruments, Tokyo, Japan) and the concentration was determined by the peak height. The lowest detectable concentrations of As(III), As(V), MMMA(V), and DMAA(V) were 0.02, 0.11, 0.18, and 0.12 nmol L^−1^ with RSD values (n = 3) of 1.3, 2.7, 2.5, and 2.3%, respectively^87^.

### Cell size

A minimum of 35 individual cells was measured in each sample using a microscope (KEYENCE, VHX-1000, Japan) at 500X magnification. Cell diameter and height were measured as they appeared on the micro slide glass, considering the cells as cylinders. Cell surface and volume were then calculated in each temperature and salinity condition.

### Statistics

Statistical analysis was carried out using SPSS 22.0 for Windows (IBM Co., USA) and Graph Pad Prism 7.0 (GraphPad Software Inc., USA). One-way and two-way analysis of variance (ANOVA) with a Tukey test was conducted to determine significant differences between the means for growth and As biotransformation of each diatom species at each temperature and salinity.

## Supplementary information

Supporting information
