# Supplementary material for: Arsenic biotransformation potential of six marine diatom species: effect of temperature and salinity
Source: Sci Rep. 2019 Jul 15;9:10226. doi: 10.1038/s41598-019-46551-8 (PMC6629621; doi:10.1038/s41598-019-46551-8)
Supplement: Supplementary file 1 — Supporting information [file 41598_2019_46551_MOESM1_ESM.docx]

**Appendix A: Supporting information**

**Arsenic biotransformation potential of six marine diatom species: effect of temperature and salinity**

Rimana Islam Papry^1,^*, Kento Ishii^1^, M. Abdullah Al Mamun^1^, Sohag Miah^1^, Kanako Naito^2^, Asami S. Mashio^3^, Teruya Maki^3^, Hiroshi Hasegawa^3,^*

*^1^Graduate School of Natural Science and Technology, Kanazawa University, Kakuma, Kanazawa-920-1192, Japan*

*^2^Department of Environmental Science, Faculty of Life and Environment, Prefectural University of Hiroshima, Shibazuka-cho, Ebara-shi, Hiroshima-727-0023, Japan*

*^3^Institute of Science and Engineering, Kanazawa University, Kakuma, Kanazawa-920-1192, Japan*

*Corresponding author (s):

papry015@gmail.com (R.I. Papry); hhiroshi@se.kanazawa-u.ac.jp (H. Hasegawa),

Tel / Fax: 076-234-4792

Table S1. Cell surface area (µm^2^) of six diatom species under different temperature and salinity condition.

| Temper-ature (°C) | ***A. karianus*** | ***T. nitzschioides*** | ***N. longissima*** | ***Skeletonema sp.*** | ***D. brightwellii*** | ***C. didymus*** |
| --- | --- | --- | --- | --- | --- | --- |
| 0 | 353±37^a^ | 683±74^a^ | 3236±398^a^ | 768±140^a^ | 3432±363^a^ | 1298±66^ac^ |
| 5.0 | 409±54^a^ | 902±84 ^a^ | 3737±378^a^ | 1221±119^b^ | 4090±384^a^ | 1592±71^ab^ |
| 10 | 524±33^ab^ | 1018±64 ^ab^ | 4187±415^a^ | 1196±162^b^ | 4114±363^ab^ | 1702±103^ab^ |
| 15 | 592±33^b^ | 1020±63 ^ab^ | 4312±462^a^ | 1316±88^b^ | 3544±277^a^ | 1715±60^ab^ |
| 20 | 594±57^b^ | 920±98^ab^ | 4950±463^ab^ | 1271±125^b^ | 3177±349^a^ | 1703±60^ab^ |
| 25 | 526±29^ab^ | 856±61^ab^ | 3873±293^a^ | 1260±102^b^ | 3137±371^a^ | 1633±190^ab^ |
| 30 | 360±41^a^ | 642±91^a^ | 3944±303^a^ | 1236±79^b^ | 3032±252^a^ | 811±119^c^ |
| 35 | 300±46^a^ | 536±52^a^ | 3923±355^a^ | 919±58^ab^ | 2696±340^a^ | 617±102^c^ |
| Salinity (‰) | ***A. karianus*** | ***T. nitzschioides*** | ***N. longissima*** | ***Skeletonema sp.*** | ***D. brightwellii*** | ***C. didymus*** |
| 0.3 | 252±55^a^ | 1005±44^a^ | 2255±113^a^ | 753±113^a^ | 3184±165^ab^ | 1754±74^ab^ |
| 1.0 | 292±22^a^ | 1057±31 ^a^ | 3677±471^b^ | 794±126^a^ | 3580±204^ab^ | 1722±88^ab^ |
| 3.5 | 399±57^a^ | 1033±63 ^a^ | 3908±157^b^ | 961±80^ab^ | 3841±292^ab^ | 1905±99^ab^ |
| 5.0 | 367±62^a^ | 968±107 ^a^ | 3374±85^b^ | 1007±88^ab^ | 4071±265^a^ | 2104±218^ab^ |
| 10 | 406±50^a^ | 1221±117^a^ | 3484±151^b^ | 1170±40^ab^ | 4112±90^a^ | 2234±244^a^ |
| 15 | 432±48^ab^ | 1127±195^a^ | 4133±287^bc^ | 1274±51^b^ | 4343±282^a^ | 2240±267^a^ |
| 20 | 499±41^ab^ | 955±42^a^ | 3945±463^bc^ | 1158±127^ab^ | 4032±221^a^ | 2154±224^a^ |
| 25 | 542±58^ab^ | 1071±199^a^ | 4242±156^bc^ | 1147±132^ab^ | 4086±251^a^ | 2260±160^a^ |
| 30 | 508±35^ab^ | 945±53 ^a^ | 4442±254^bc^ | 1111±195^ab^ | 4118±158^a^ | 2064±258^ab^ |
| 35 | 369±68^a^ | 755±91^ab^ | 4373±282^bc^ | 901±114^ab^ | 3788±299^ab^ | 1846±223^ab^ |
| 40 | 307±43^a^ | 670±34^b^ | 3762±283^b^ | 844±65^a^ | 3920±332^ab^ | 1604±143^ac^ |
| 45 | 326±35^a^ | 6403±63^b^ | 3924±298^b^ | 865±152^a^ | 3017±237^ab^ | 1273±128^c^ |
| 50 | 264±40^a^ | 548±42^b^ | 3278±406^b^ | 797±145^a^ | 2827±387^ab^ | 1172±194^c^ |

Different lowercase letter indicates significant differences between temperature and salinity levels *(p* <0.05). Data are means ±SD (*n*=3).

Table S2. Composition of artificial seawater^1^.

| Compounds | Weight |
| --- | --- |
| MgCl_2_ · 6H_2_O | 108 g |
| NaCl | 240 g |
| NaHCO_3_ | 1.96 g |
| KCl | 6.8 g |
| Na_2_SO_4_ | 40.00 |
| NaF | 0.029 g |
| H_3_BO_3_ | 0.250 g |
| SrCl · 6H_2_O | 0.122 g |
| KBr | 1.00 g |
| CaCl_2_· 6H_2_O | 14.3 g |
| Purified water |  |
| Total | 10 L |

References

1 Lyman, J. & Flemming, R. Composition of seawater. *J Marine Res III* **2**, 134-146 (1940).
